# Temperament and Attention-Deficit/Hyperactivity Disorder: A Narrative Review on Developmental Risk and Clinical Implications

**DOI:** 10.1192/j.eurpsy.2026.11134

**Published:** 2026-07-31

**Authors:** M. D. Pereira, J. Torres, C. Bayam, S. Neiva

**Affiliations:** Pedopsiquiatria, Hospital Pediátrico de Coimbra, Coimbra, Portugal

## Abstract

**Introduction:**

Attention-deficit/hyperactivity disorder (ADHD) is a common neurodevelopmental condition that typically emerges in early childhood. Temperament refers to biologically based individual differences in emotional reactivity, attention and self-regulation, observable from infancy and relatively stable across development. Core dimensions, such as negative affectivity, surgency/extraversion and effortful control, have been extensively investigated in relation to later psychopathology. Emerging evidence suggests that early temperament profiles may confer vulnerability to ADHD and shape its symptom trajectory and clinical expression.

**Objectives:**

To review recent evidence on the association between early childhood temperament traits and the development of ADHD, with focus on identifying risk patterns, temperament-based subtypes and their implications for early clinical intervention.

**Methods:**

We performed a narrative review of studies published in the last five years, using PubMed with the search terms “temperament”, “attention-deficit/hyperactivity disorder” and “childhood”. Included studies were longitudinal or cross-sectional and examined associations between early temperament traits and ADHD symptomatology or diagnosis in paediatric populations.

**Results:**

Studies consistently indicate that high activity level, negative affectivity, and low effortful control are associated with increased ADHD risk. Longitudinal findings suggest these traits can precede and predict ADHD symptom trajectories. Temperament-based subgroups, such as high surgency versus high negative affectivity, are linked to different comorbidity profiles, including oppositional and anxiety symptoms. While methodological heterogeneity exists, a converging body of evidence supports the dimensional role of temperament in early ADHD vulnerability.

**Conclusions:**

Temperament traits offer a clinically useful framework for identifying children at increased risk of ADHD. Incorporating temperament assessment into psychiatric evaluation may support early detection and guide personalised, developmentally informed intervention strategies. Further research should explore how temperament-based models can contribute to clinical decision-making and risk stratification.

**Disclosure of Interest:**

None Declared

